# Using the Online Psychotherapy Tool to Address Mental Health Problems in the Context of the COVID-19 Pandemic: Protocol for an Electronically Delivered Cognitive Behavioral Therapy Program

**DOI:** 10.2196/24913

**Published:** 2020-12-18

**Authors:** Nazanin Alavi, Megan Yang, Callum Stephenson, Niloofar Nikjoo, Niloufar Malakouti, Gina Layzell, Jasleen Jagayat, Amirhossein Shirazi, Dianne Groll, Mohsen Omrani, Anne O'Riordan, Sarosh Khalid-Khan, Rafael Freire, Elisa Brietzke, Fabiano Alves Gomes, Roumen Milev, Claudio N Soares

**Affiliations:** 1 Department of Psychiatry Faculty of Health Sciences Queen's University Kingston, ON Canada; 2 Centre for Neuroscience Studies Faculty of Health Sciences Queen's University Kingston, ON Canada; 3 School of Rehabilitation Therapy Faculty of Health Sciences Queen's University Kingston, ON Canada; 4 Centre for Behavioural Studies St. Lawrence College Kingston, ON Canada; 5 OPTT Inc Digital Media Zone Ryerson University Toronto, ON Canada; 6 Kingston Health Sciences Centre Kingston, ON Canada; 7 Inpatient Psychiatry Unit Kingston General Hospital Kingston Health Science Centre Kingston, ON Canada

**Keywords:** mental health, COVID-19, depression, anxiety, psychotherapy, cognitive behavioural therapy, online, internet, electronic, mental health care

## Abstract

**Background:**

The considerable rise of mental health challenges during the COVID-19 pandemic has had detrimental effects on the public health sector and economy. To meet the overwhelming and growing demand for mental health care, innovative approaches must be employed to significantly expand mental health care delivery capacity. Although it is not feasible to increase the number of mental health care providers or hours they work in the short term, improving their time efficiency may be a viable solution. Virtually and digitally delivering psychotherapy, which has been shown to be efficient and clinically effective, might be a good method for addressing this growing demand.

**Objective:**

This research protocol aims to evaluate the feasibility and efficacy of using an online, digital, asynchronous care model to treat mental health issues that are started or aggravated by stressors associated with the COVID-19 pandemic.

**Methods:**

This nonrandomized controlled trial intervention will be delivered through the Online Psychotherapy Tool, a secure, cloud-based, digital mental health platform. Participants will be offered a 9-week electronically delivered cognitive behavioral therapy program that is tailored to address mental health problems in the context of the COVID-19 pandemic. This program will involve weekly self-guided educational material that provides an overview of behavioral skills and weekly homework. Participants (N=80) will receive personalized feedback from and weekly interaction with a therapist throughout the course of the program. The efficacy of the program will be evaluated using clinically validated symptomology questionnaires, which are to be completed by participants at baseline, week 5, and posttreatment. Inclusion criteria includes the capacity to consent; a primary diagnosis of generalized anxiety disorder or major depressive disorder, with symptoms that started or worsened during the COVID-19 pandemic; the ability to speak and read English; and consistent and reliable access to the internet. Exclusion criteria includes active psychosis, acute mania, severe alcohol or substance use disorder, and active suicidal or homicidal ideation.

**Results:**

This study received funding in May 2020. Ethics approval was received in June 2020. The recruitment of participants began in June 2020. Participant recruitment is being conducted via social media, web-based communities, and physician referrals. To date, 58 participants have been recruited (intervention group: n=35; control group: n=23). Data collection is expected to conclude by the end of 2020. Analyses (ie, linear regression analysis for continuous outcomes and binomial regression analysis for categorical outcomes) are expected to be completed by February 2021.

**Conclusions:**

If proven feasible, this care delivery method could increase care capacity by up to fourfold. The findings from this study can potentially influence clinical practices and policies and increase accessibility to care during the COVID-19 pandemic, without sacrificing the quality of care.

**Trial Registration:**

ClinicalTrials.gov NCT04476667; https://clinicaltrials.gov/ct2/show/NCT04476667

**International Registered Report Identifier (IRRID):**

DERR1-10.2196/24913

## Introduction

### Background and Rationale

The COVID-19 pandemic is a source of high-degree uncertainty, anxiety, and stress, and it is ultimately affecting mental health on a global scale. According to a recent survey, 75% of Canadians have reported feeling anxious, 37% have reported feeling lonely, and 32% have said that they are having a hard time falling asleep because of stressors associated with the COVID-19 pandemic [1]. Previous experience has shown that the psychological scars resulting from public disasters go far beyond the official end of the disaster. For instance, the mental health effects that were observed following Hurricane Katrina lasted for over 4 years after the disaster, with a 35% increase in substance abuse hospitalization in New Orleans [2]. This shows that addressing the mental health aspect of the COVID-19 pandemic is as important as addressing the immediate medical emergency. The increase in demand for mental health care services resulting from the COVID-19 pandemic has come in the backdrop of a system that is already in crisis, which necessitates devising innovative approaches for increasing the current care capacity to cover more patients.

Cognitive behavioral therapy (CBT) could be a promising solution for treating depression and anxiety disorders related to the COVID-19 pandemic. CBT is widely regarded as a first-line treatment for major depressive disorder (MDD) and generalized anxiety disorder (GAD), as supported by randomized controlled trials, meta-analyses, and the recommendations of most clinical guidelines [3-7]. CBT has proven to be effective in relieving depressive and anxiety-related symptoms, improving the overall functioning of patients with such symptoms, and preventing relapses of these conditions [3-5], with long-term effects [6,7]. However, effectively administering CBT through face-to-face or virtual/live (ie, synchronous) methods is very time consuming and costly, and in most cases, such methods are not accessible to many patients. Electronically delivered CBT (ie, nonlive, asynchronous care; e-CBT) could be a viable alternative method for mental health care delivery. E-CBT has proven to be efficacious in treating both anxiety and depressive disorders, with comparable results to in-person therapy [8-11]. E-CBT content can be conveniently accessed anytime and anywhere, thereby providing flexible access to care, even for people who live in remote areas. A major benefit of e-CBT is that by delivering predesigned therapy content, clinicians can skip repeating similar general concepts to multiple patients and reduce their time commitment to a fraction of that in in-person therapy [12,13]. This not only reduces the cost of care delivery, but also allows more people to receive care from existing clinical resources and shortens waiting times. The main disadvantage to using e-CBT as a treatment delivery modality is that some individuals might not have access to the necessary technology (eg, phones, tablets, or laptops) or proper internet connectivity. Furthermore, there are different methods of delivering e-CBT, which range from unguided self-help [14,15] to guided programs that consist of a standardized content delivery modality with support from a mental health professional [12,13,16]. Although these different methods have been shown to be effective to some extent, their efficacy varies depending on the level of care provider engagement during the therapy process [17]. Naturally, as the level of clinical engagement goes higher, so does the cost of therapy, thereby making the therapy less accessible to patients in need. We believe a hybrid solution that combines predesigned content with limited and personalized guidance from a clinician could lower the costs of therapy, increase care capacity, and improve patient outcomes through increased patient engagement. Given the structured nature of CBT, even clinician feedback could follow a predesigned structure to make the process more streamlined.

In this protocol, we aim to develop a scalable online psychotherapy clinic centered around e-CBT to assist patients with stress resulting from the COVID-19 pandemic. The e-CBT program consists of 9 weekly modules that focus on coping skills and building resilience, which are effective in the treatment of mood and anxiety disorders. We will use the Online Psychotherapy Tool (OPTT; OPTT Inc) [18], a secure, cloud-based platform, to interact with patients and deliver therapy. By using the OPTT, all patient information can be kept secure and confidential, while allowing all clinicians on the treatment team to access their patients’ information and communicate with one another through the platform to discuss their patients. We hypothesize that using these psychotherapeutic interventions during the COVID-19 pandemic will improve the quality of life and decrease symptoms of depression and anxiety in the intervention group compared to the control group.

### Objectives

The first objective of this study is to design and implement a scalable online psychotherapy clinic and care plan (ie, content, design, and feedback structure plans) to address mental health problems that started or worsened during the COVID-19 pandemic. The second objective is to evaluate the feasibility and efficacy of treating COVID-19–related mental health problems by offering specific e-CBT modules through an online clinic. The third and final objective is to rapidly disseminate the knowledge gained from this study to other practices, thereby facilitating the effective and reliable scaling of this solution across the community.

## Methods

### Study Design

This clinical trial has a nonrandomized controlled trial design. Participants in the intervention group will be offered a 9-week e-CBT program that is tailored to address mental health problems in the context of the COVID-19 pandemic. Qualitative focus groups will be conducted to gather personal demographic information, as well as information about the feasibility of implementing an online psychotherapy clinic. Additionally, quantitative analyses of online psychotherapy treatment efficacy will be conducted using standardized symptomology questionnaires. All procedures have been approved by the Queen’s University Health Sciences and Affiliated Teaching Hospitals Research Ethics Board.

### Participants

Participants (N=80) aged 18-65 years will be enrolled in the study based on referrals from the Hotel Dieu Hospital and Providence Care Hospital outpatient clinics located in Kingston, Ontario, Canada, and self-referrals from social media and web-based communities. Those referred and interested in participating will provide informed consent before being evaluated by one of the psychiatrists on the research team through a secure video appointment. During this appointment, MDD/GAD diagnoses will be made or confirmed using the Diagnostic and Statistical Manual of Mental Disorders, 5th Edition (DSM-5). Mini-International Neuropsychiatric Interview, Version 7.0.2 DSM-5 will also be administered by a trained research assistant on the team to confirm the diagnosis. Inclusion criteria includes the capacity to consent; a primary diagnosis of GAD or MDD, with symptoms that started or worsened during the COVID-19 pandemic; the ability to speak and read English; and consistent and reliable access to the internet. Exclusion criteria includes active psychosis, acute mania, severe alcohol or substance use disorder, and active suicidal or homicidal ideation. Additionally, to keep the scope of this study focused on the more common challenges that people face during the pandemic, participants will be excluded if mental health problems are secondary to a medical condition. Moreover, if a participant is currently receiving or has received CBT in any format during the past year, they will be excluded to prevent a possible confounding effect between previous CBT treatment and the e-CBT program.

Participants who are eligible for this study will be offered to join the electronic psychotherapy (e-psychotherapy) group. If individuals in this treatment group have been on pharmacotherapy treatment, their medication from 6 weeks prior to the trial and during the 9 weeks of treatment will not get changed. Individuals diagnosed with MDD or GAD who do not wish to participate in the e-psychotherapy group will continue to receive treatment as usual (TAU) and will be part of the control group. TAU is defined as any lifestyle activities and interventions (ie, medication, psychiatric consultation and referrals, exercise, diet, etc) that participants in either group were receiving prior to joining the trial. These activities and interventions will not be changed throughout the course of participants’ participation. Both groups will be stratified by sex, gender, and age.

### Procedures

Participants enrolled in the e-psychotherapy group will participate in a 9-week program that will include a combination of CBT, mindfulness therapy, and problem-based therapy. The content of the e-psychotherapy program will be customized to reflect the challenges that individuals may face during the COVID-19 pandemic and developed into interactive and engaging therapy modules. A sample session has been provided by OPTT Inc [19]. All online sessions and interactions will occur through a secure, online platform called the OPTT.

Each participant will be assigned to a therapist on the research team. During each week, the therapist will assign a predesigned therapy module to their patient through the OPTT on a specific day of the week. Participants will have access to the therapy content at any time throughout the week. Each weekly module will focus on a new topic by covering general information on the topic, providing an overview of the skills associated with the topic, and providing homework, which will be due on a specific day of the week. All content and topics that will be covered are designed to mirror in-person CBT programs. Completing each weekly module will require an average time commitment of 40 minutes, which can be completed all at once or in several blocks of time. The homework will be directly submitted through the OPTT to the clinician, who will then provide personalized feedback to the patient.

In order to maintain care and outcome consistency and streamline the process, therapists will use predesigned session-specific feedback templates to respond to each homework submission. This will also help to save time and costs and make the program more scalable. Therapist feedback will generally follow a certain structure. First, the therapist will start off feedback by valuing the participant’s time and effort. This will help to establish a rapport between the therapist and the participant. Second, the therapist will summarize the previous sessions’ material and remind the participant of what they have learned so far. The information in this section will differ between sessions, but it will remain constant across participants. Third, the therapist will discuss the participant’s homework and provide an evaluation. The therapist will always review the event that the participant used in their homework, so that the participant will know that their therapist has read and paid attention to their unique situation. Fourth, the therapist will discuss what the participant did right and what the participant could have done differently in their homework. Last, the therapist will emphasize their appreciation for the participant’s hard work and sign their name. This will ensure that the connection between the therapist and the participant remains personal. Writing feedback using this structure usually does not take longer than 15-20 minutes for an experienced therapist. A more detailed explanation on this structure can be found in Online Cognitive Behavioral Therapy: An e-Mental Health Approach to Depression and Anxiety, a book by Alavi and Omrani [20].

Although the homework and clinician feedback are considered the main means of communication between therapists and participants, participants can also communicate with their therapist through a secure chat function that is found directly within the OPTT. This is mainly used to let participants ask further questions about their care, if anything is not clear. Any technical issues will be handled through the OPTT technical support team, which participants will have access to at all times during the program. The patient care team (eg, the therapist and the psychiatrist) will also be able to securely communicate through the OPTT to make decisions regarding each patient’s care path.

The control group will continue receiving TAU for the first 9 weeks of their participation. If control group participants continue to present significant symptoms (ie, <50% reduction in symptoms compared to those in baseline), they will again be offered the option to undergo the e-psychotherapy program or other forms of treatments as deemed necessary.

### Online Modules

The modules are designed for individuals who experience symptoms of anxiety and depression in the context of COVID-19 pandemic. We combined our expertise in content development [20] from previous clinical trials, which used a similar approach to address depression [21] and anxiety [12], and certain content from previous therapeutic modules, and adapted them to address COVID-19–related stressors and develop relevant strategies. We then discussed the content with an expert panel of therapists, psychiatrists, and end users to ensure the suitability and efficiency of the content. During this program, therapists will help participants understand that in any given situation, their thoughts, feelings, and actions all interact with and influence one another and help them change their thoughts and actions. The goal of this program is to teach individuals how to identify and change their destructive and disturbing thought patterns, which have a negative influence on behavior and emotions. Through these modules, we will discuss the effect of the pandemic on mood, the basics of CBT, deep breathing techniques, body scan and meditation, the self-care kit, SMART (Specific, Measurable, Achievable, Realistic, and Timely) goals, thinking errors, 5-part models, and thought records. The first 2 sessions will be designed to address symptoms caused by the fear of illness and concerns about personal safety in the context of the pandemic. The rest of the sessions will focus on cognitive and behavioral techniques, problem-solving techniques, and mindfulness practices to help build healthy coping skills that address the uncertainties surrounding the COVID-19 pandemic and the symptoms of depression and anxiety. During this program, we will focus on essential thinking and behavioral skills to help individuals become more engaged in day-to-day activities. We will also work on evaluating negative beliefs and thought processes, as well as their relationship with anxiety/depression. Our goal is to adjust negative thinking so that participants can think about and adapt to the things that are happening to them. This will allow participants to adjust the way they behave, think about their problems in a way that is not as negative, and replace negative thoughts with potentially more positive and productive thoughts and behaviors.

In addition to the content, we pay special attention to the presentation of materials to participants. There are multiple animations and interactive examples that will be provided in each session to keep the participants interested and engaged, such as those in the OPTT Inc sample session [19]. The modules are accessible with any device (ie, desktops, tablets, and cellphones) and compatible across multiple browsers.

### Training

All the therapists are research assistants hired by the research team. These therapists have previous training in psychotherapy, and they will receive further training from one of the psychiatrists involved in the study before they interact with participants. Therapists will learn the standard care pathway, aim, and content of each therapeutic session. Moreover, they will be provided with sample homework from patients and asked to provide feedback as practice for each session. As explained earlier, the feedback templates will vary from session to session, and therapists will personalize each template for each patient’s homework. Training will be provided through webinars and exercises with feedback. Therapists will also be supervised by the lead psychiatrist on the team, who has more than 10 years of experience in in-person and online psychotherapy. After the feedback is prepared by the therapist, the feedback will be read, edited, and approved by the supervisor before it is submitted to the participant.

During the consent process, participants are informed that this program is not to be used as a crisis resource, as therapists cannot be reached at all times through the OPTT platform. In the case of an emergency (eg, suicidal ideation), therapists will be instructed to direct patients to the proper resources (eg, emergency department, crisis lines, etc) and inform their supervisor about such incidents.

### Ethics and Data Privacy

All procedures have been approved by and comply with the Queen’s University Health Sciences and Affiliated Teaching Hospitals Research Ethics Board. Only the care providers involved in the care of the participant will have access to their participant’s information. Hard copies of consent forms and participant identity will be securely stored on-site and destroyed 5 years after study completion. Participants will only be identifiable by an ID number on the OPTT platform, and only anonymized data will be provided to the analysis team members.

To ensure data privacy and security, the care platform (ie, the OPTT) was developed so that it complies with the Health Insurance Portability and Accountability Act, Personal Information Protection and Electronic Documents Act, and Service Organization Control-2. All servers and databases are hosted in the Amazon Web Service Canada cloud infrastructure, which is managed by Medstack (Medstack Inc) [22], to ensure that all Canadian provincial and federal privacy and security regulations are met. For privacy purposes, the OPTT will not collect any identifiable personal information or internet protocol addresses from participants. The OPTT will only collect anonymized metadata to improve its service quality and provide advanced analytics data to the clinical team. All data will be encrypted by the OPTT, and no employee will have direct access to participant data. All encrypted backups are to be kept in the Amazon S3 storage that is dedicated to Queen’s University, Kingston, Ontario, Canada.

### Outcome Evaluation

Primary outcome measures will be anxiety level based on Generalized Anxiety Disorder-7 (GAD-7) Item Questionnaire scores, depression level based on Patient Health Questionnaire-9 Item scores, resilience level based on Resilience Scale-14 Item Questionnaire scores, and quality of life based on Quality of Life and Enjoyment Questionnaire scores. All questionnaires will be collected directly through the OPTT at baseline, prior to week 5, and after the last session.

In addition to these quantitative measurements, qualitative analyses will be performed to evaluate the efficacy and feasibility of providers delivering e-CBT and participants receiving e-CBT. Health care providers will be asked about the feasibility of providing e-psychotherapy and how it compares to in-person psychotherapy in terms of time commitment, feelings of connectedness to the participant, and any perceived benefits and drawbacks to e-psychotherapy. Information on personal, social, and cultural factors (eg, gender, sexuality, background, supportive resources, structural/social barriers, etc) will be extracted from focus groups using an interpretive phenomenological analysis approach. Additionally, participants will be asked about their experiences with using the OPTT and their positive or negative experiences with the e-CBT program. Program adherence and completion rates will also be recorded and reported.

### Data Analysis

Initially, all data will be examined for missing, nonsensical, and outlying variables. Missing data will be treated as missing and not imputed. This will be analyzed on a per-protocol basis. Given the likelihood of participant drop out or withdrawal, we have purposely oversampled our study and control groups to obtain meaningful and statistically significant results at the end of the study. Based on our previous experience with CBT and e-CBT in similar patient populations, we anticipate that up to 30% of participants will drop out by the end of the treatment or TAU phases. Using GAD-7 questionnaire scores as the primary outcome means that a 30% change is considered clinically significant. Therefore, a sample size of 40 participants in each arm of the study will be sufficient for detecting significant results (ie, *P*=.05 and a power of 0.95). In addition, an intention-to-treat analysis will be performed to evaluate completion rates and the clinical effects of participants leaving the trial before completion.

Data collection will occur at 3 separate time points, as follows: at baseline (ie, preintervention), in the middle of the study (ie, week 5), and immediately at postintervention (ie, week 9). Mann-Whitney U tests will be used to compare baseline demographic data between individuals who drop out and those who finish the e-CBT program, and any fundamental differences between completers and noncompleters will be identified. A linear regression analysis for continuous outcomes and binomial regression analysis for categorical outcomes will be performed to identify variables associated with the 2 outcome measures over the 3 measurement time points and to compare the differences between study arms. These analyses will be controlled for demographic variables, such as age and gender.

## Results

The study was approved for funding in May 2020. Additionally, ethics approval was received from the Queen’s University Health Science and Affiliated Teaching Hospitals Research Ethics Board in June 2020, and the recruitment of participants began in June 2020. Participant recruitment has been based on physician referrals and self-referrals through social media and web-based communities. To date, 58 participants have been recruited (ie, 35 in the intervention group and 23 in the control group). Data collection is expected to conclude by the end of 2020, and analyses are expected to be completed by February 2021. We will share the outcomes of our study as a preprint on bioRxiv to rapidly disseminate our findings. We will also hold multiple online workshops for other clinicians who are interested in implementing this approach. During these workshops, we will provide technical and academic support to other clinicians so that they can deploy this solution in their respective practices.

## Discussion

Addressing the extensive mental health problems resulting from the COVID-19 pandemic requires rapid and easily accessible solutions. An online psychotherapy clinic with predesigned therapy modules can be used to rapidly scale up clinical capacity to address mental health problems resulting from the COVID-19 pandemic, while also ensuring a high quality of care. That is why, through this study, we emphasize developing and validating a comprehensive solution (ie, engaging and effective content, feedback templates, and clinician training) that can be easily deployed with minimal resources. We are also planning to disseminate the outcomes of our study through publicly accessible media, so other clinicians can easily access and incorporate this solution into their clinical practices in time to meet their patients’ needs. This approach will result in major financial savings for the health care system, as it provides methods for the efficient use of clinicians’ time and the equitable and accessible delivery of care to patients.

It should be noted that our current study design has some limitations, such as the nonrandomized design of the study. In order to address these concerns in a structured format, we are using the “guidance for reporting intervention development studies in health research (GUIDED)” framework [23] and “template for intervention description and replication (TIDieR)” [24] to report our protocols and outcomes (Tables S1 and S2 in Multimedia Appendix 1). We are also planning to start a randomized study in January 2021. Additionally, the electronic mode of care delivery might not be suitable for specific subgroups of patients (eg, older adults or patients who lack experience with related technologies). We hope that through our qualitative focus groups, we will be able to identify each of these subgroups and find suitable remedies for future iterations of our study design (eg, changing the module design so that it is user-friendly for older adults).

Delivering care through a digital platform provides new opportunities for tracking behavior in a way that was not possible before [25], such as new methods for tracking sleep patterns and activity level changes across multiple people with mood disorders. Currently, subjective and qualitative descriptions of sleep and activity changes are used for tracking patients’ mood changes. However, with the widespread use of wearable devices that can quantitatively track these behaviors, incorporating such devices in patients’ care could provide an invaluable source of information for monitoring patient progress.

In order to take advantage of this new avenue, the new randomized study in January 2021 will incorporate the procedure explained above. In this study, each participant will be provided a wearable device that will record their activities and sleep patterns. Data from the wearable devices will be automatically synched and collected with the OPTT platform, and analytical data will be provided to clinicians. An additional session will be added to the current modules to provide information regarding sleep hygiene and the importance of remaining active during the pandemic. Clinicians will also monitor participants’ sleep patterns and activities for significant changes (ie, increases or decreases in sleep duration and decreases in activity), provide feedback to the participants, and encourage participants to follow a healthy routine. We believe that this comprehensive plan will significantly boost the positive effect of our e-CBT program.

## Appendix

Multimedia Appendix 1 Reporting Guidelines.

## Abbreviations

CBT: cognitive behavioral therapy
DSM-5: Diagnostic and Statistical Manual of Mental Disorders, 5th Edition
e-CBT: electronically delivered cognitive behavioral therapy
e-psychotherapy: electronic psychotherapy
GAD-7: Generalized Anxiety Disorder-7
GUIDED: guidance for the reporting of intervention development
MDD: major depressive disorder
OPTT: Online Psychotherapy Tool
SMART: specific, measurable, achievable, realistic, and timely
TAU: treatment as usual
TIDieR: template for intervention description and replication
